# An Easy Death

**Published:** 1857-06

**Authors:** 


### AN EASY DEATH.

NOT the least of all the rewards of a life of systematic temperance, is that of an easy death. The whole machinery of the body wears out together. Its fly-wheels and its rollers, its cogs, its scapements, and its springs, lose all their power by equal and slow degrees. No one part runs on in the full vigor of its newness, while others are wholly incapacitated. "He suffered a thousand deaths in his last illness," is the familiar description of the closing scene of many. And why? Because one part of the complicated machinery had worn out before its time, from having been overtasked, or had been made a wreck of, by destructive habits or exposures. It is the being "*temperate in all things*" to which the sacred Scriptures attach the blessing of the life that now is, as well as of that which is to come: to which we may allowably attach the meaning, enjoyment of to-day, exemption from suffering on to-morrow. Present health and an easy death are the uniform perquisites of those who obey the Scripture injunction in the love of it.

No less a violation of the inflexible law of our being is it to wear out the throat by vociferous preaching; or the voice organs by injudicious singing; or the brain by ruthless habits of mental appliances; or the eyes by persistence in night study; or the imagination by unlicensed delving into the "*hidden wisdom*" in order to be wise *above that which is written;* or the stomach by taxing it daily with a labor it was never formed to accomplish; or the hands themselves or feet by imposing a task on their capabilities which they were never made to endure; we say these are no less infractions of physical law than are wilful violations of written moral precepts. As to the latter, we have an "Advocate" who can "clear" us; from the former no power can deliver, short of the miraculous, and that, it is useless to expect.

It is the regular and temperate who live long. It is the very old who die without sickness or pain—whose lamp of life goes out as gently as the last flicker of an expiring candle. *Cornaro* died at ninety-six, without the illness of a day. Old *Aunt Hay* died among the nineties, without the sickness of an hour. The Rev. Mr. Davies, of England, had no disease of any kind during his life except poor sight, and died at the age of a hundred and five years.

If, then, we covet an "*easy death*" as to the body, let us obey the *Book* of books in being

### TEMPERATE IN ALL THINGS.

And more, if we would "*die easy*" as to the more immortal part, the soul, let us still cling to the guardianship of that sacred volume, and be like *Cornelius*, men "*without guile*," striving "*to have always a conscience void of offence toward God and toward men.*"

---

IN A HURRY.—"Doctor, mother sent me down to the shoticary pop quicker'n blazes, cos bub's sick as the dickens, with the picken chox, and she wants a thimbleful of pollygolic in this din tipper, cos we ha'nt bot a gottle, and the kint pup's got the bine witters in't. Got any?"

